# Helping Caregivers to be Ready, Willing, Able, and Healthy

**DOI:** 10.1093/geroni/igaa057.2399

**Published:** 2020-12-16

**Authors:** Robyn Golden, Vikki Rompala, Ellen Carbonell

**Affiliations:** 1 Rush University Medical Center, CHICAGO, Illinois, United States; 2 Rush University Medical Center, Chicago, Illinois, United States

## Abstract

Given extended life expectancy, family caregivers are needed to provide care for older adults at home. Research has documented the deleterious effects of caregiving on mental and physical health for many. The Caregiver Health and Well-Being Initiative is a systems approach to support family caregivers of older adults by standardizing processes for identifying caregivers in ambulatory and inpatient settings, assessing caregivers’ needs, and providing relevant services and resources, including caregiver health services, a Teach Back clinic for skills development, and extended goals of care conversations. N=104 caregivers have completed assessments. Participants reported the following at baseline: depressive symptoms (54%); anxiety symptoms (69%); health being affected by the care situation (59%); financial situation decreasing (58%); feeling torn between demands of their family and demands of care (63%); and some feelings of burden from caregiving (97%). Intervention components will be discussed along with a larger systems change framework for implementation.

